# “*We are not ready for this*”: physicians’ perceptions on climate change information and adaptation strategies - qualitative study in Portugal

**DOI:** 10.3389/fpubh.2024.1506120

**Published:** 2024-12-17

**Authors:** Nidia Ponte, Fátima Alves, Diogo Guedes Vidal

**Affiliations:** ^1^Centre for Functional Ecology - Science for People & the Planet (CFE), TERRA Associate Laboratory, Department of Life Sciences (DCV), University of Coimbra, Coimbra, Portugal; ^2^Department of Social Sciences and Management, Universidade Aberta, Lisbon, Portugal; ^3^Sergio Arouca National School of Public Health (Ensp), Oswaldo Cruz Foundation (Fiocruz), Rio de Janeiro, Brazil

**Keywords:** climate change and health, public health policy, physician perception, healthcare professionals, climate resilience, health vulnerabilities, health adaptation strategies

## Abstract

**Background:**

Climate change presents several challenges to public health and its professionals. This article aims to fill a significant gap in the current literature by understanding physicians’ perceptions of their role in educating others about health adaptation to climate change. It also explores their knowledge of health policies related to this issue in Portugal and their perceived influence on the development of adaptation policies at both local and national levels within the health sector.

**Methods:**

To this end, we applied a qualitative and case study approach, interviewing 13 physicians in Portugal, including general practitioners and specialists. The data was collected using a semi-structured interview script, and a content analysis was performed to categorize the responses and gain a comprehensive understanding of the phenomenon.

**Results:**

The main results of this study highlight the need for a more systematic approach to training physicians, including the relationship between climate change and health. Concerning policies, this research highlights the need for more consistent communication and precise guidelines for dealing with the impacts of climate change on public health.

**Conclusion:**

As the first exploratory study focusing on Portuguese physicians, this research provides unique insights into their views on the potential to influence patient behavior and health policy. Importantly, it offers valuable recommendations for health policy strategies, particularly in awareness-raising and training plans for these professionals, thereby demonstrating the research’s practical implications.

## 1 Introduction

The health impacts of climate change (CC) are becoming increasingly severe as global temperatures rise. This urgent situation needs for policy responses to mitigate these effects and protect public health. The Intergovernmental Panel on Climate Change (IPCC) predicts that in the 21st century, it will be a challenge to limit the increase in temperature to 2°C if immediate mitigation measures are not taken (1, 2). In this context, the role of public health policy is critical in shaping interventions that address the health consequences of CC, particularly by involving healthcare professionals and communities in evidence-based strategies (3). However, as seen in the various Conferences of the Parties^1^, achieving effective political and policy actions remains challenging despite growing public and scientific awareness. Different social groups, particularly young^2^ people, whose futures are at risk, have increasingly challenged the inertia and ineffectiveness of political actions.

The intersection of climate change and health equity is exceptionally pressing. Vulnerable populations, such as children, the older adult, the chronically ill, and socio-economically disadvantaged people, are particularly at risk (4, 5). The IPCC (1) report identifies climate change as a driver of increased poverty and accentuated inequalities, exacerbating pre-existing health problems. Portugal, a southern European country with a Mediterranean climate, stands uniquely susceptible and vulnerable to health risks resulting from climate change due to its geographical location and the socio-economic characteristics of its population (6).

In response to CC, individuals’ cognitive and emotional involvement is central and is affected by behavioral changes and civic and political activities (7). Several studies have emphasized the perceptions of CC at the population level, that is, how they explain and deal with this phenomenon daily (8, 9). While the health impacts of CC have been extensively studied, much of the existing research has focused on clinical outcomes, epidemiological patterns, and disease surveillance (10). For instance, Alves, Leal, and Vidal (6) warn that central and southern Portugal are areas that could suffer from the appearance of insects that transmit diseases such as Dengue, Malaria, and the Zika virus (which exist in tropical climates), thus significantly affecting public health. This presents significant challenges for public health, calling for policy-driven preparedness and mitigation strategies.

Given this, health professionals have an essential role in formulating strategies adapted to specific socio-ecological environments to reduce the impact of CC vulnerabilities and increase the resilience of populations (11, 12). Moreover, because they are well-informed and trustworthy, they are in a solid position to communicate the risks posed by CC and the benefits of adaptation (13). In addition, they can influence the organizations where they work to reduce their emissions (14). Also, Campos et al. (15) states the importance of physicians’ involvement in the fight against CC, stating that it is physicians’ ethical obligation to become actively involved in the fight against CC and environmental degradation. Similarly, André et al. (16) recognize that as an outreach physician, the family physician must actively raise awareness among their patients, influencing behavioral change by giving advice that benefits the individuals and planetary health.

Thus, our motivation for this topic arises because we recognize the significant role of physicians in response to CC, as they are the first to deal with the health consequences of CC. Also, they may influence the preparation of health systems for the health impacts of CC, as well as change the behavior of their patients and public opinion. In this context, studying physicians’ perceptions of CC in the context of the ecological transition is justified by the relevance that these professionals have in terms of public health. This research can provide valuable insights that inform health policy recommendations toward a healthier and more sustainable future for all, underlining the influential role of physicians in this critical issue.

This is more relevant since studies on health professionals are scarce, especially about physicians’ perceptions of CC. The existing quantitative studies (17–24) have primarily focused on measuring the level of awareness and concern among physicians about CC. In contrast, our quality study aims to delve deeper into their perceptions and experiences by applying semi-structured interviews. Also, our study complements other research that also uses a qualitative methodology (12, 25, 26) and systematic review (27, 28) by contributing to the literature on this subject and by presenting the first exploratory study of physicians in a Portuguese context that provides clues for outlining recommendations for health policy strategies, particularly about awareness-raising and training plans for these professionals.

Recognizing the pivotal role of physicians in the context of CC (11, 13, 15, 29), we focused this analysis on the perceptions of a group of physicians with clinical practice in Portugal, with the utmost respect for their expertise and insights. Our primary objective is to understand whether they perceive themselves as critical players in raising awareness among their patients about the impacts of CC on health, broken down into the following specific objectives:

- Characterize how physicians perceive their role in adapting to climate change health impacts.
- Identifying physicians’ information on CC policies in health.
- To understand whether these professionals believe their actions will likely influence local and national policies to adapt to climate change in the health sector.

## 2 Methodology

Our study is grounded in a robust qualitative methodology (30), which allows us to understand the reality experienced by individuals, considering their perceptions, values, representations, beliefs, opinions, attitudes, and habits. We have chosen the case study strategy (31) to define boundaries and circumscribe the phenomenon within its context, enabling an in-depth analysis of its various aspects. Our decision to conduct this research was informed by a literature review that revealed a lack of empirical research on physicians’ contributions to public awareness and policy engagement regarding CC. Therefore, our exploratory study (32) seeks to address this gap, offering valuable insights that can inform future public health policy strategies, particularly around physician involvement in climate change adaptation and mitigation efforts.

### 2.1 Data collection

We used semi-structured interviews, which allowed the interviewees to express their perceptions and experiences while providing an in-depth understanding of the analyzed phenomenon. Also, it will enable the interviewer to guide the conversation, and it has the flexibility to explore topics brought up by the participants that had not been considered before. To respond to this research’s objectives, we drew up a script with open questions (see Table 1 and the original text in Supplementary material 1). When drawing it up, we considered other studies carried out on physicians’ perceptions and which successfully applied their questionnaires and interviews (24–26). We conducted a script test to ensure that the questions were well formulated, i.e., that our interviewee understood them and that they would enable us to achieve our objectives. Since the questions were open, the participants were free to interpret the scope of the inquiry, allowing us to delve into their perceptions and experiences. This approach facilitated a comprehensive understanding of the phenomenon under analysis. We also applied a questionnaire to characterize the participants demographically (Supplementary material 2).

**Table 1:** Semi-structured interview guide.

Questions	Guiding questions
1st Objective - Characterize how physicians perceive their role in adapting to climate change health impacts.	
1st category: role of physicians	
Do you think it’s important for physicians to address CC issues and their impact on health with their patients?	Why? How do you think they could do it?
Have you ever broached the subject of CCs in a conversation with your patient?	If so, why did you do it?
	What advice did you give to change habits that could improve your health and mitigate the effects of CC?
	If not, why and if so, are you planning to do so?
Do you think physicians can influence their patients to change some of their behaviors to benefit their health and the environment?	Why?
Do you think physicians can influence the public and policy-makers to be more proactive in taking more effective measures to combat CC?	How? No Why?
Do you think physicians can play an active role in encouraging those responsible in their workplace to be as environmentally sustainable as possible?	How?
2nd and 3rd objectives - Identifying physicians’ information on CC policies in health. To understand whether these professionals believe their actions will likely influence local and national policies to adapt to climate change in the health sector.	
2nd category - CC health policies	
Do any government bodies provide information about illnesses, or their exacerbations related to CC?	For example: National Health Services, Ministry of Health, Central, Regional or local health administration
Are there procedures in place? At what level? What are they? And how do you think it should work?	Do you think there should be this kind of information?
In your opinion, are the health services prepared to deal with the aggravations that CC brings to human health? Why?	What are your suggestions for improving this issue?
In your opinion, who do you think is responsible for finding solutions to the consequences of CC?	

Source: authors own work.

In the final week of September 2022, we initiated a meticulous process to establish initial contact via email with local health units in Porto, Portugal, to identify physicians willing to participate. This correspondence yielded contact information for relevant individuals, to whom we promptly dispatched all pertinent details to facilitate their decision-making regarding participation in our research. Recognizing the challenge of engaging physicians, we also encouraged interviewees to suggest other colleagues for inclusion in the study, employing a technique known as snowball sampling (32). This comprehensive approach, leveraging both provided contacts and technological capabilities, enabled us to successfully recruit participants from three distinct districts. Out of the 20 physicians contacted, 16 responded. Unfortunately, two participants did not appear at the scheduled interviews, and one had to be canceled due to technical issues with the recording. The selection criteria, which included availability, willingness to participate, and active medical practice in Portugal, were designed to ensure the reliability and diversity of our study. Since the primary focus of this study was on physicians practicing medicine in Portugal, the only exclusion criterion was not practicing medicine in Portugal.

The interviews took place between October 12 and November 2, 2022. The authors have considerable experience conducting qualitative research and interviews, but the corresponding author conducted each interview to ensure consistency and rigor. Before each interview, we reiterated the purpose of the research and obtained oral and written consent from the participants to record the interview. For the participants’ convenience, interviews took place in person (2 at the Senhora da Hora Health Centre), by telephone (7), and by videoconference (4 Zoom or Jitsi Meet). The meetings lasted an average of 30 min, the shortest being 17 min (face-to-face) and the longest 43 min (Zoom). The participants were 13 physicians practicing medicine in Portugal, 8 females and 5 males, aged between 28 and 73. Table 2 shows demographic details.

**Table 2:** Demographic data of the participants in the study.

Code	Sex	Age	Medical specialty	Years in the profession	Type of workplace		District
E1	F	29	General and Family Medicine - Internal	4	Healthcare Center	Public	Porto
E2	F	37	Reconstructive Plastic Surgery	12	Hospital	Public	Porto
E3	F	46	General and Family Medicine	22	Healthcare Center	Public	Porto
E4	F	29	General and Family Medicine - Internal	3	Healthcare Center	Public	Porto
E5	F	73	Occupational Medicine	43	Private Company	Private	Aveiro
E6	M	69	Internal Medicine	44	Hospital	Private	Porto
E7	M	48	Pulmonology	23	Hospital	Private	Porto
E8	M	55	Pulmonology	30	Hospital	Public	Coimbra
E9	F	33	General and Family Medicine- Internal	3	Healthcare Center	Public	Porto
E10	F	28	General and Family Medicine - Internal	2	Healthcare Center	Public	Aveiro
E11	M	39	General and Family Medicine	7	Healthcare Center	Public	Porto
E12	F	35	Internal Medicine	10	Hospital	Public	Porto
E13	M	45	Internal Medicine	21	Hospital	Private	Porto

We had 8 women (61.5%) and 5 men (31.5%). This aligns with national statistics, which show more women than men in the medical profession. Indeed, according to the Portuguese Medical Association, 57% of women and 43% of men are in the medical profession.

Source: authors own work.

### 2.2 Data analysis

We used content analysis (33) to interpret the interviews in this study, guided by an abductive approach (Table 3), which facilitated iterative engagement between the data, theoretical framework and research objectives. The process began with transcribing the interviews using Microsoft Word 365 and carefully reviewing the transcripts for accuracy. This initial review, instrumental in gaining a comprehensive understanding of the content, laid a solid foundation for the subsequent steps. We then conduct multiple readings to facilitate the systematic coding of phrases, words, and concepts relevant to our research questions. These codes were categorized based on the participants’ responses and the study’s objectives. The iterative nature of the abductive approach allowed us to revisit theoretical assumptions as recurring patterns or unexpected themes emerge during the analysis. This process involved examining similarities, differences and overarching patterns across the data. Codes were grouped into broader themes that represent the core ideas arising from the participants responses. Themes were then reviewed and refined to reflect participant’s perspectives accurately while aligning with the study’s theoretical orientation. We conducted the entire analysis using MAXQDA 2020 (48), a robust and reliable qualitative analysis tool, which streamlined the process and ensured methodological rigor. To enhance inter-rater reliability and credibility, we implemented a systematic and transparent approach for resolving disagreements in establishing codes and themes. The MAXQDA Logbook allowed us to document coding rules, changes, and clarifications. At the same time, the Teamwork Comparison Tool facilitated the analysis of discrepancies. Regular meetings were held to discuss challenging cases, update the coding framework, and recalibrate as necessary. Table 3 expresses the main categories and subcategories that emerge in this process.

**Table 3:** Categories resulting from the content analysis of the interviews.

Category	Specific objectives	Subcategories
Role of physicians	Characterize how physicians perceive their role in adapting to climate change health impacts.	Address CC and its impact on health with their patients in consultation
		If they think they can influence patients to change their behavior
		Whether and how they can influence the general public
		Whether or how they influence behavioral change about CC in the workplace
CC health policies	Identify physicians’ information on CC policies in health and understand whether these professionals believe their actions will likely influence local and national policies to adapt to CC in the health sector.	They consider whether or not their role can influence policymakers to draw up CC adaptation and mitigation policies.
		Whether they receive information from public bodies and the government about the health impacts of CCs
		Whether or not public services are prepared to deal with health impacts and respond to needs
		Whose responsibility, is it?

Source: authors own work.

We enhanced the credibility of our findings by employing several strategies. First, we used triangulation by collecting data through semi-structured interviews and demographic questionnaires, which provided multiple perspectives on the phenomenon under study. This approach ensured that our conclusions were comprehensive and well-rounded. Additionally, our interviews were conducted until theoretical saturation was reached, ensuring no new themes emerged and strengthening our conclusions’ validity. The consistency in interview procedures, managed by the corresponding author, also contributed to the study’s credibility by minimizing interviewer bias. Member checking, where participants were asked to review and confirm their interview transcripts’ accuracy, further reinforced our interpretations’ credibility. While our study is context-specific, focusing on physicians practicing in Portugal, we aimed to ensure transferability by clearly describing the research context, participant characteristics, and the phenomenon under study. This detailed contextualization allows other researchers to determine how our findings may apply in different contexts or with similar populations.

To ensure dependability, we maintained an audit trail documenting every step of the research process, from data collection to analysis and interpretation. This included detailed records of interview protocols, coding procedures, and theme development, all systematically recorded using the MAQDA 2020. The consistency of the research process was also supported by the involvement of experienced qualitative researchers, ensuring the research was conducted with high methodological rigor. Finally, we addressed confirmability by ensuring that our findings were derived directly from the data and not influenced by researcher bias or personal perspectives. This was achieved through reflexivity, where the research team continually reflected on their potential biases and how they might affect the research process.

### 2.3 Ethics

Regarding ethics, this project followed all the standards required by the Open University for qualitative research and was approved by the scientific council. As a result, all participants were given prior access to the information necessary and relevant to their decision to participate via the “Information” form (Supplementary material 3). Before each interview, participants read and signed the “Informed Consent” document (Supplementary material 4).

## 3 Results

In this section, we present the findings of our study, beginning with a characterization of the study group (3.1) and then the results. During the content analysis, two broad categories emerged related to the specific objectives (see Table 3). We have used these categories to organize this section: (3.2) the role of physicians, where we characterize how physicians perceive their role in adapting to climate change health impacts; and (3.3) health climate change policy aims to identify physicians’ information on CC policies in health and to understand whether these professionals believe their actions will likely influence local and national policies to adapt to climate change in the health sector.

### 3.1 Characterization of the participants

Our 13 respondents were between 28 and 73 years old, 8 females and 5 males. The participants’ levels of education ranged from a bachelor’s degree to a PhD. The participants came from different specialties, but most were general, and family practitioners and the majority had more than 10 years of work experience. The majority also work in the public sector (six work in health centers and three in hospitals), while the remaining three work in private hospitals and one in a company. Table 2 shows the details of the demographic characterizations of the interviews.

### 3.2 The role of physicians

None of the physicians interviewed said they had discussed CC and its health impacts with their patients. Despite this, 8 interviewees said they ask their patients to change their behavior, but always from the point of view of lifestyle and health promotion, without mentioning CC.

*Yes, for health promotion, but we do not talk about climate change [E3, F, 46 General and Family Medicine].*

*Not directly, with climate change [E2, F, 37, Reconstructive Plastic Surgery].*

In contrast, 5 respondents said they had never approached their patients about changing their behavior. They explained that either they needed more professional experience and had not had the chance to experience this situation, or the subject had never come up because they worked in palliative care and had other priorities.

*Not really, but my experience over the last 3 years has been somewhat limited [E4, F, 29, General and Family Medicine].*

*I work in palliative care, so I think many other priorities exist [E13, M, 45, Internal Medicine].*

Given these answers, we wanted to find out what obstacles prevented them from talking with patients to change their behavior. The most significant difficulty pointed out by the interviewees was the length of the consultation. They use their time to listen to and diagnose the pathology presented by the patient at that moment. Another reason was the patient’s availability, i.e., physicians can give advice, but patients may not be willing to act on it, either because they do not want to or because of the influence of others, such as television programs, friends, or neighbors.

*Another thing is that we have time to do it, but no, we do not have time to do it because we have very short appointments [E11, M, 39 General and Family Medicine].*

*It depends on the person’s motivation because we can say, “Look, do this, do that, do that,” and then they will go home and do nothing [E3, F, 46 General and Family Medicine].*

Even so, all the participants recognized the importance of including the causes and consequences of CC on health in the conversation between physicians and patients. They stated that alerting people to their actions is vital, as they can affect their health and contribute to CC. It is, therefore, crucial to advise and educate people on the best way to act and protect themselves.

*It also helps patients realize that they can help prevent climate change through their daily lives and attitudes, their health, and, above all, those who have risk diseases [E4, F, 29, General and Family Medicine].*

*Certainly, therefore, this is fundamental, especially in more vulnerable populations. General and family medicine and pulmonology, which are the specialties that are probably most at the forefront of this area, play a very special role here [E8, M, 55, Pulmonology].*

*I think so in the context of primary health care. To enlighten people, right? [E12, F, 35, Internal Medicine].*

Some interviewees also highlighted the lack of training for health professionals on the relationship between CC and its impact on health.

*We are not ready for this (pause). There should be a course in college [E3, F, 46 General, and Family Medicine].*

*So, we need to understand climate change, what we can do to avoid or mitigate it, and, above all, how we can include it in our clinical practice [E7, M, 48, Pulmonology].*

Twelve participants admit that physicians can be a good source of information regarding the influence they can exert on their patients to change their behaviors. Some interviewees mentioned that, in the case of the general and family medicine specialty, it is part of these physicians’ job to accompany people throughout their lives, establishing a relationship of trust and thus being able to induce a behavior change. In this sense, several interviewees mentioned that they try to advise their patients on healthy lifestyle habits.

*I think we have to, at least if it’s beneficial, we have to at least try to influence them towards healthy lifestyle habits, do not we? [E9, F, 33, General and Family Medicine].*

*In our specialty, we accompany people throughout their lives; we establish a crucial doctor-patient relationship with most people, and with this relationship, we influence them through our behavior, too, do not we? So, yes, we pass on information; we have this capacity to influence [E11, M, 39 General and Family Medicine].*

However, 2 of the participants believe that in their role as physicians, they do not have the power to influence their patients to change their behavior, stating that:

*In that respect, I think being a physician does not count. Yes, let me give you two very classic examples: only those who want to stop smoking and only those who want to lose weight [E6, M, 69, Internal Medicine].*

*I find that very difficult. It is practically a losing battle. If I change one patient out of 100, I will not have much impact; I will have minimal impact [E7, M, 48, Pulmonology].*

On the other hand, all participants agreed that they could influence the organizations where they work to be more proactive in CC mitigation and adaptation measures. Some of the attitudes that participants mentioned, by way of example, are the encouragement to use electronic tools more frequently to reduce paper consumption and the recycling of other items, such as coffee capsules and hospital waste.

*For example, my team consumes coffee and recycles its capsules [E12, F, 35, Internal Medicine].*

*At the hospital, the physicians put pressure on the administration regarding managing some hospital waste [E13, M, 45, Internal Medicine].*

Finally, 9 interviewees agree that they can influence the public to be more active in the fight against CC because physicians are responsible for health and have the necessary scientific knowledge. However, 4 say physicians have less authority and a lower position of respect to influence the public. They also stated that nowadays, people give more importance to what they see, for example, on television programs or even to what specific influencers say, such as Greta Thunberg than to what is advised or transmitted by physicians.

### 3.3 Health climate change policies

Our interviewees’ speeches reveal a lack of consensus on the information or guidance they receive from public services about CC and its impact on health. Half acknowledge official communication on these issues, while the other half deny its existence. Those who receive information do so through maps or group meetings, focusing on extreme events like heat waves and cold wave. Those who claim to not receive official information highlight their reliance on mass media for updates.

*In our hospital, information can occasionally come through, but it is very occasional. There is no newsletter or information circular that makes a regular note, you know? [E13, M, 45, Internal Medicine].*

*As far as the medical side is concerned, nothing is said, no procedure is issued, and no instructions are given on how to proceed with these situations [E4, F, 29, General and Family Medicine].*

*I think we receive information in the summer about heat waves and what to do, but not specifically about climate change, right? [E10, F, 28, General and Family Medicine].*

*It’s a map of the time of the heatwave and then the winter cold wave, and they’ll give you some guidance [E11, M, 39 General and Family Medicine].*

*For example, when the weather changes, whether it is heat or cold waves, right? Physicians do not receive any warnings apart from the ones they hear about in the media [E13, M, 45, Internal Medicine].*

All 12 participants unanimously stress the importance of regular and comprehensive information, including current events and additional knowledge. This information is crucial for addressing the health challenges their patients face, particularly in the context of CC. Only 1 participant holds a different view, stating.

*“I am not a consumer of this kind of information” [E1, F, 29, General and Family Medicine].*

Generally speaking, the interviewees who mentioned the importance of having more information about CC and health mainly mentioned information about the health risks that could affect their patients and who the most vulnerable and at-risk groups are.

*So, to identify the patients who are most at risk, they will be the most vulnerable, but there may be things that do not happen, right? [E10, F, 28, General and Family Medicine].*

*So, would it make sense from the point of view of those on the other side? It would be relevant to understand if there is anything in the next few days that could be a health risk for the patients. I think it is essential to be attentive to patients [E13, M, 45, Internal Medicine].*

The study aimed to understand how participants perceive the National Health Service’s (NHS) ability to manage the increasing impacts of CC on human health. Most interviewees (8:13) expressed that the NHS is not adequately equipped to deal with the worsening effects of CC on health, highlighting the urgent need for better preparation within the healthcare system.

*We are putting more pressure on the system than it would be chaotic, as there is neither the capacity to respond nor sufficient resources [E12, F, 35, Internal Medicine].*

*I suppose not. One reason is that climate change is already happening, and the structure and functioning of health services are the same as they were 20 years ago or 30 years ago. (…) Most hospitals and clinics with infections that can come from climate change are not well prepared for this, nor are the physicians trained or experienced [E7, M, 48, Pulmonology].*

However, some interviewees (3:13) believe the NHS can effectively respond to disaster situations.

*I suppose there are two levels of consequences of climate change. One is disasters, such as floods, in which health services intervene to resolve the situation and care for the victims. From the point of view of new microbes and agents, I think that our health services are prepared and are responding in line with what I said earlier [E6, M, 69, Internal Medicine].*

Finally, one respondent did not answer (1:13). Another expressed more ambivalent positions (3:13): that there is some awareness of this issue and even mentions the existence of plans for extreme events; however, defending that there is still much work to be done because these events will become increasingly frequent.

*I think we can cope as long as they are very specific situations. I think it will be a bit of a stretch in more severe situations, as it was with COVID [E4, F, 29, General and Family Medicine].*

Regarding the improvements the NHS would need to respond to the future worsening situations that CC could cause, the most mentioned was training for health professionals to be prepared to act and inform their patients (9:13). The relationship between CC and medical practice should be explained. The necessary information about deviations from the norm should be provided so that they can treat patients. Another improvement mentioned is to strengthen primary health care, with an increase in hiring professionals and giving them incentives to stay in the NHS. They emphasized the need for proactive prevention strategies, prioritizing prevention more.

*The first thing we need to do is integrate everything related to climate change and link it to medical practice [E7, M, 48, Pulmonology].*

*Have a department that reports on this and that it is only about this and that we give us support and if we need any more in-depth training [E5, F, 73, Occupational Medicine].*

Another dimension we analyzed with our respondents was physicians’ ability to influence policymakers to develop more effective CC mitigation and adaptation policies. More than half (7:13) demonstrate their disbelief that they, as professionals, can pressure policymakers regarding CC.

*They state that politics in Portugal “is not much influenced by physicians” [E4], indicating that “political decision-makers are rarely on the side of science and evidence” [E7].*

Even so, 4 participants said it was possible to exert influence on politicians, not as physicians and individuals, but as part of an organization, such as the Physicians’ Association, showing the strength of the collective. Here is one of the excerpts that illustrates this opinion:

*These exhibitions, changing laws, have to be in conjunction with the Ministry of Health and the Order of Physicians, for example [E2, F, 37, Reconstructive Plastic Surgery].*

From another point of view, 2 interviewees say that the only influence they can exert is on an individual level, through elections, because by choosing a government party, they elect a program that may or may not include CC policies.

Finally, in Table 4, we look at the participants’ perceptions in this research regarding the responsibility to find the solutions for mitigation and adaptation to CC. Seven interviewees say that individuals and politicians must get involved in this fight because the small steps taken can be reflected globally if everyone moves in this direction. However, if policymakers create policies that facilitate and encourage individual actions, the result will be even more significant. From another perspective, 3 participants say that it is partly up to the individual to take action and that it is up to the citizen to pressure politicians. Another 2 said that both politicians and scientists are responsible for knowledge and the implementation of guidelines and, therefore, must set an example and encourage people and companies to act to combat CC. Alongside the previous answers, they also mentioned large companies and environmental associations.

**Table 4:** The responsibility for finding solutions to the consequences of CC?

Who’s responsible	No. of occurrences
Politicians and individuals	7
Individuals	3
Big companies	3
Politicians and scientists	2
Environmental associations	2

Each respondent recognized multiple items (multiple responses).

Source: authors own work.

## 4 Discussion

As far as we know, this is the first attempt in Portugal to describe and understand whether physicians identify themselves as critical actors in making their patients aware of the health impacts of CC. Our findings, reveal that 11 participants (the majority) recognize that physicians can be powerful catalysts for change. They can influence their patients and workplaces to alter their behavior, potentially leading to significant health and environmental improvements. However, additional knowledge and education are crucial to translate this belief into action. Providing physicians with the necessary tools and understanding will empower physicians to confidently recognize CC as a health issue and take adequate measures to address it. They can leverage their influence as health professionals and through their organizations (such as the Order of Physicians) to raise awareness before the general public. In terms of influencing political decision-makers, seven interviewees expressed dissatisfaction with the political class, suggesting that they can only impact CC policies through organized efforts. This finding underscores the need for a policy framework that empowers physicians and health professionals to engage in CC mitigation and adaptation, with structured mechanisms for their involvement in decision-making processes.

In this research, physicians emphasize the need to discuss lifestyle choices and their impact on patients’ health to raise awareness about behaviors that may affect their health and contribute to CC. For instance, they have started advising patients on the health benefits of walking or cycling instead of driving or the importance of reducing meat consumption for both personal health and the environment. However, some factors can hinder these conversations during consultations. One such factor is the limited time available, typically used for listening and diagnosing the disease. Another obstacle is the lack of knowledge and preparation required to engage in these conversations. Additionally, patient motivation plays a role, as some patients may be unwilling to engage in these discussions. Despite these challenges, some participants have already begun addressing these issues with their patients, focusing on health behaviors without mentioning CC.

Our results reinforce previous studies outside Portugal and found in the literature review on physicians’ perceptions of this topic (17, 19, 25). These studies show physicians believe they can be essential in CC mitigation strategies. They also show that there is a need for training, with Sanderson & Galway (26) and Boer (25) pointing to a lack of training as an obstacle to communicating about the links between CC, the environment, and health, as well as how they can communicate these links without damaging the doctor/patient relationship. They also point out that CC is described as a politicized issue, representing a further obstacle. This underscores the need for the development of public health that offer training programs to equip physicians with skills and resources to effectively address climate change impacts on health.

Portugal aligns its environmental policies with those of the European Union. The country has put in place various mechanisms to reduce greenhouse gas emissions. There is an increasing emphasis on adaptation measures involving non-governmental entities in decision-making processes for CC policies and strategic document submissions for public consultation (34–36). In addition, researchers highlights communication challenges regarding CC, emphasizing the need for improved education to enhance communication strategies (37–39).

Our research, however, shows that some measures regarding the impacts of CC on health need to be improved. This is where government bodies and public health policies can play a crucial role—by providing physicians with more regular information and training, focusing on the link between CC and health, and offering measures to apply in their practice so that they can pave the way for significant improvements. Aligning with this, the participants in this research recommend that the NHS disseminate regular information that addresses current and future events and contributes other types of knowledge. For instance, they suggest sharing information about the impact of dust coming from North Africa and the best practices to address it. Additionally, they proposed improvements to the performance of the NHS, particularly in situations with an exacerbation due to CC. They also emphasized the importance of training (a recurring theme at almost every point in this analysis), strengthening primary care, hiring professionals and incentives to keep them in the NHS, and the need to prioritize prevention.

Regarding the capacity of the NHS to deal with the health problems that CC can cause, nine participants said that the system does not have enough resources and is not equipped for such an eventuality. They gave the example of COVID-19, pointing out that if what happened in Italy had occurred in Portugal, things would have been much worse; there would have been many more deaths, and it would have been chaotic. This underscores the potential for CC to exacerbate existing health challenges and create new ones and the urgent need for the healthcare system to be prepared.

Finally, seven participants (more than half) stated that individuals and politicians must fight against CC. In a study carried out in 2011, Schmidt and Delicado (40) found that more than half of the participants believed that the responsibility for combating CC is collective. In our survey, if we only count those who mentioned the individual as responsible for fighting CC, we will get a much higher figure than the one above. This could indicate an increase in individual responsibility for adopting pro-environmental behaviors. Barr et al. (41) predicted this shift in responsibility. They stated that CC policies made individuals more responsible than economic actors or public administrations. However, it is essential to note that the government, as highlighted in a recent survey by Leal e Filho et al. (42), also plays a crucial role in implementing regulations and policies to effectively address the public health hazards of CC, reassuring us about the collective effort in combating climate change. Still, as Alves et al. (3) mentioned, the responsibility must be shared, and it should be considered not only by the local government and police but also by the community’s lived experience.

In summary, the need for more knowledge and training to link CC to health and to broach the subject in a conversation with their patients was highlighted at various interviews. Moreover, these health professionals are already overburdened by their workload, with consultations lasting very short, just long enough to diagnose the patient. However, the physicians’ role as educators and influencers of changes in individual behavior is recognized, so training and support are urgently needed. On the other hand, the NHS currently needs to be able to respond to the health problems that CC can cause. Currently, there are only a few CC policies in the health sector, mainly focused on extreme temperatures.

Furthermore, the National Health Plan 2021–2023 categorizes the health impacts of CC as a problem of ‘low or no magnitude, but with a high-risk potential’ (43), only recently mentioning health professionals on its website. However, the World Health Organization (44) asserts that a resilient health system relies on well-trained staff and sufficient resources. Therefore, it is crucial to develop training that empowers health professionals to engage in climate communication and advocacy effectively. Moreover, we should reframe the narrative to underscore climate action as an opportunity to enhance individual and public health, fostering a sense of optimism and possibility.

## 5 Limitations

One of the limitations we have faced since the beginning was the scarcity of bibliography and scientific literature on the perceptions of CC with a focus on health professionals, namely physicians. However, some Portuguese works at the national level emphasize social perceptions on CC (9, 45–47). This study employs a qualitative methodology and a case study approach, as it aims to explore a relatively under-researched topic. Consequently, we focused on capturing the diversity of perceptions rather than seeking their regularity. The primary objective was to map these varied perceptions without attempting to quantify or generalize them. This approach inherently limits the study’s ability to provide statistically generalizable results. However, it allows for a deeper understanding of the nuances and complexities of the subject matter. Moreover, this research makes it possible to draw some conclusions about the participants’ perceptions of CC in the defined context. It is also possible to draw some recommendations for health policy strategies, specifically regarding awareness-raising and education plans for these professionals.

## 6 Conclusion

CC significantly impact human health, such as an increase in respiratory and cardiovascular diseases, a worsening of allergies and asthma, an increase in the incidence of vector-borne diseases such as Malaria and Dengue, and a decrease in food and water security. In this context, physicians are critical, as they are on the front line responding to the effects of CC on health and must, therefore, have the necessary knowledge to recognize the origins of these diseases and to engage in the fight against anthropogenic CC, as they pose a severe threat to human health. Therefore, health professionals, particularly physicians, are central to developing preventive, promotional, and remedial strategies for the causes and consequences of CC on human health. Within this framework, our research explores physicians’ potential to raise awareness among their patients and influence climate policies.

This research reveals the pivotal role that physicians recognize they play in raising patient awareness about the health impacts of CC. They believe they can influence their patients and workplaces to adopt healthy behaviors and actions to mitigate and adapt to CC. However, they also expressed the need for more knowledge and institutional support to deal effectively with this issue. Importantly, they highlighted the significant role of physicians’ organizations and collective actions in influencing CC-related policies, thereby emphasizing the importance of physicians in shaping climate policies.

This study’s most significant contribution is the insight it provides into the perspective of physicians, a professional group about which we have had limited knowledge until now. Understanding their perceptions is crucial, given the substantial influence they can have in promoting changes in behavior among their patients and in shaping public opinion. The study also highlights the state of CC policies for health in Portugal, underscoring the need for more proactive measures, particularly those that involve health professionals. A robust health system hinges on a sufficient number of well-trained professionals with adequate resources. Therefore, there is a pressing need for additional training to establish the relationship between CC and health and raise awareness among health professionals.

We conclude that researching physicians’ and other health professionals’ knowledge regarding the health effects of CC is essential to preparing healthcare services. These professionals can help create rapid strategies to change individual behaviors, which are necessary for the context of ecological transition and in minimizing some of the effects of global warming. Equally relevant is the contribution to a change in narrative that emphasizes CC mitigation and adaptation as an opportunity that benefits both public and individual health.

## Data Availability

The original contributions presented in the study are included in the article/Supplementary material, further inquiries can be directed to the corresponding author.

## Footnotes

^1^The Conferences of the Parties (COPs) are global meetings to discuss climate change issues. They began in 1997 in Kyoto under the name COP3 and have been held annually ever since.

^2^For example, the famous Greta Thunberg is an international example, and in Portugal the movements “Greve Climática Estudantil Portugal” and “Movimento GAIO.”

## References

[1] IPCC. Physical science basis. Contribution of working group I to the sixth assessment report of the intergovernmental panel on climate change. Cambridge: Cambridge University Press (2021).

[2] Lee H Romero J IPCC. (2023). Climate change 2023: Synthesis report summary for policymakers. Geneva, Switzerland: IPCC. 1–34.

[3] Alves F Caeiro S Azeiteiro UM. Special issue on “lay rationalities of climate change”. Int J Climate Change Strategies Manag. (2014) 6:1756–8692. doi: 10.1108/IJCCSM-10-2013-0121

[4] Alves F Leal C. Saúde humana In: Loureiro J Alves F Castro P Figueiredo A, editors. Plano Intermunicipal de Adaptação às Alterações Climáticas da CIM Região de Coimbra. Coimbra: Comunidade Intermunicipal da Região de Coimbra (2017)

[5] Watts N Adger WN Agnolucci P Blackstock J Byass P Cai W. Health and climate change: policy responses to protect public health. Lancet. (2015) 386:1861–914. doi: 10.1016/S0140-6736(15)60854-6 26111439

[6] Alves F Leal C Vidal D. Doenças transmitidas por vetores num contexto de alterações climáticas: Antecipando riscos para uma melhor preparação dos territórios. Estudo de caso da região de Coimbra. Recima21. (2023) 4:e4104181. doi: 10.47820/recima21.v4i10.4181

[7] Wolf J Moser SC. Individual understandings, perceptions, and engagement with climate change: insights from in-depth studies across the world. WIREs Clim Change. (2011) 2:547–69. doi: 10.1002/wcc.120

[8] Morgado F Bacelar-Nicolau P Rendon von Osten J Santos P Bacelar-Nicolau L Farooq H. Assessing university student perceptions and comprehension of climate change (Portugal, Mexico and Mozambique). IJCCSM. (2017) 9:316–36. doi: 10.1108/IJCCSM-08-2016-0123

[9] Schmidt L. (2018). Alterações climáticas: percepções, envolvimento e governança.,” Atas das XX jornadas sobre ambiente e desenvolvimento - Alterações climáticas e a região de Leiria - Desafios e oportunidades. OIKOS - Associação de Defesa do Ambiente e do Património da Região de Leiria. Available at: https://repositorio.ul.pt/bitstream/10451/40303/1/ICS_LSchmidt_ALteracoes.pdf (Accessed October 5, 2022).

[10] Tavares A. O impacto das alterações climáticas na saúde. Acta Medica Port. (2018) 31:241–2. doi: 10.20344/amp.10473, PMID: 29916353

[11] Winkler MS Röösli M Ragettli MS Cissé G Müller P Utzinger J. Mitigating and adapting to climate change: a call to public health professionals. Int J Public Health. (2015) 60:631–2. doi: 10.1007/s00038-015-0722-7, PMID: 26227490

[12] Ponte N Alves F Vidal DG. Exploring Portuguese physicians’ perceptions of climate change impacts on health: a qualitative study. J Clim Chang Health. (2024) 20:100333. doi: 10.1016/j.joclim.2024.100333 PMC12851290 41647127

[13] Frumkin H McMichael AJ. Climate change and public health. Am J Prev Med. (2008) 35:403–10. doi: 10.1016/j.amepre.2008.08.019, PMID: 18929964

[14] Gill M Stott R. Health professionals must act to tackle climate change. Lancet. (2009) 374:1953–5. doi: 10.1016/S0140-6736(09)61830-4, PMID: 19942275

[15] Campos L Barreto JV Bassetti S Bivol M Burbridge A Castellino P. Physicians’ responsibility toward environmental degradation and climate change: a position paper of the European Federation of Internal Medicine. Eur J Intern Med. (2022) 104:55–8. doi: 10.1016/j.ejim.2022.08.001, PMID: 36055953

[16] André H Gonzalez Holguera J Depoux A Pasquier J Haller DM Rodondi P-Y. Talking about climate change and environmental degradation with patients in primary care: a cross-sectional survey on knowledge, potential domains of action and points of view of general practitioners. Int J Environ Res Public Health. (2022) 19:4901. doi: 10.3390/ijerph19084901, PMID: 35457768 PMC9029888

[17] Hawkins IW Balsam AL Goldman R. A survey of registered dietitians’ concern and actions regarding climate change in the United States. Front Nutr. (2015) 2:21. doi: 10.3389/fnut.2015.00021, PMID: 26217666 PMC4495332

[18] Kotcher J Maibach E Miller J Campbell E Alqodmani L Maiero M. Views of health professionals on climate change and health: a multinational survey study. Lancet Planet Health. (2021) 5:e316–23. doi: 10.1016/S2542-5196(21)00053-X 33838130 PMC8099728

[19] Kreslake JM Price KM Sarfaty M. Developing effective communication materials on the health effects of climate change for vulnerable groups: a mixed methods study. BMC Public Health. (2016) 16:946. doi: 10.1186/s12889-016-3546-3, PMID: 27604549 PMC5015239

[20] Majra J Acharya D. Protecting health from climate change: preparedness of medical interns. Indian J Community Med. (2009) 34:317–20. doi: 10.4103/0970-0218.58390, PMID: 20165625 PMC2822192

[21] Pescosolido BA Martin JK JD ML Rogers A. Handbook of the sociology of health, illness, and healing: a blueprint for the 21st century. New York, NY: Springer New York (2011).

[22] Sarfaty M Mitchell M Bloodhart B Maibach E. A survey of African American physicians on the health effects of climate change. Int J Environ Res Public Health. (2014) 11:12473–85. doi: 10.3390/ijerph111212473, PMID: 25464138 PMC4276625

[23] Sarfaty M Bloodhart B Ewart G Thurston GD Balmes JR Guidotti TL. American Thoracic Society member survey on climate change and health. Ann Am Thorac Soc. (2015) 12:274–8. doi: 10.1513/AnnalsATS.201410-460BC, PMID: 25535822 PMC5466202

[24] Sarfaty M Kreslake JM Casale TB Maibach EW. Views of AAAAI members on climate change and health. J Allergy Clin Immunol Pract. (2016) 4:333–5.e26. doi: 10.1016/j.jaip.2015.09.018, PMID: 26703816

[25] Boer AD. Physician’s role in addressing the issue of climate change and health, during their conversation with patients: a qualitative study. Wageningen, Holanda: Wageningen University & Research (2016).

[26] Sanderson R. Health professionals and climate change communication: an exploratory study in northern Ontario. Ontário, Canadá: Lakehead University (2021).

[27] Hathaway J Maibach EW. Health implications of climate change: a review of the literature about the perception of the public and health professionals. Curr Environ Health Rep. (2018) 5:197–204. doi: 10.1007/s40572-018-0190-3, PMID: 29423661 PMC5876339

[28] Buse CG Allison S Cole DC Fumerton R Parkes MW Woollard RF. Patient- and community-oriented primary care approaches for health in rural, remote and resource-dependent places: insights for eco-social praxis. Front Public Health. (2022) 10:867397. doi: 10.3389/fpubh.2022.867397, PMID: 35692331 PMC9178183

[29] Watts N Amann M Arnell N Ayeb-Karlsson S Beagley J Belesova K. The 2020 report of the lancet countdown on health and climate change: responding to converging crises. Lancet. (2021) 397:129–70. doi: 10.1016/S0140-6736(20)32290-X, PMID: 33278353 PMC7616803

[30] Creswell JW. Research design: Qualitative, quantitative, and mixed methods approaches. Los Angeles, CA: SAGE Publications (2014).

[31] Merriam S. Case studies as qualitative research In: Conrad CF Haworth JG Page L, editors. Qualitative research and case study applications in education. San Francisco: Jossey-Bass (2007). 26–43.

[32] Denzin NK Lincoln YS. Handbook of qualitative research. Los Angeles, CA: SAGE Publications (2018).

[33] Bardin L. Análise de conteúdo. Lisboa: Edições 70 (1977).

[34] Abbott D Wilson G. Climate change: lived experience, policy and public action. Int J Clim Change Strateg Manag. (2014) 6:5–18. doi: 10.1108/IJCCSM-04-2013-0040

[35] Schmidt L Delicado A Junqueira L. Políticas de alterações climáticas em Portugal: posicionamentos e redes de relações dos atores institucionais. Anál Soc. (2021) 3:470–97. doi: 10.31447/as00032573.2021240.03

[36] Mendonça A Leal Filho W Alves F. Public participation and climate change governance: between political approach and local actors’ perspective in two Macaronesian territories. Front Environ Sci. (2023) 11:178. doi: 10.3389/fenvs.2023.1094178

[37] Leal FW. Communicating climate change: challenges ahead and action needed. Int J Clim Change Strateg Manag. (2009) 1:6–18. doi: 10.1108/17568690910934363

[38] Schmeltz MT Ganesh C. Improving the capacity and diversity of local public health workforce to address climate impacts to health through community partnerships and problem-based learning. Front Public Health. (2023) 10:1090129. doi: 10.3389/fpubh.2022.1090129, PMID: 36743166 PMC9891370

[39] Ogunseitan OA. Broad spectrum integration of climate change in health sciences curricula. Front Public Health. (2022) 10:954025. doi: 10.3389/fpubh.2022.954025, PMID: 35958832 PMC9357998

[40] Schmidt L Delicado A. Ambiente, alterações climáticas, alimentação e energia: a opinião dos Portugueses In: Schmidt L Delicado A, editors. Imprensa de Ciências Sociais. Lisbon: ICS - Social Sciences Press (2014)

[41] Barr S Gilg A Shaw G. Citizens, consumers and sustainability: (re)framing environmental practice in an age of climate change. Glob Environ Chang. (2011) 21:1224–33. doi: 10.1016/j.gloenvcha.2011.07.009

[42] Leal Filho W Ternova L Fayyaz MM Abubakar IR Kovaleva M Donkor FK. An analysis of climate change and health hazards: results from an international study. Int J Clim Change Strateg Manag. (2022) 14:375–98. doi: 10.1108/IJCCSM-08-2021-0090

[43] DBP Santos. (2020). Plano Nacional de Saúde 2021-2030. Saúde Sustentável: de todos para todos. Available at: https://pns.dgs.pt/files/2022/03/PNS-21-30_Versao-editada-1_Final_DGS.pdf (Accessed November 10, 2022).

[44] WHO. Operational framework for building climate resilient health systems. Geneva: World Health Organization (2015).

[45] Rocha CLP. (2015). Perceções sociais dos portugueses face aos riscos associados às alterações climáticas. Available at: https://ria.ua.pt/bitstream/10773/15155/1/Perce%c3%a7%c3%b5es%20sociais%20dos%20portugueses%20face%20aos%20riscos%20associados%20%c3%a0s%20altera%c3%a7%c3%b5es%20clim%c3%a1ticas.pdf (Accessed February 12, 2023).

[46] Ventura AC. Antropologia do Ambiente. Um contributo para o estudo das alterações climáticas. Entre os discursos, as percepções dos riscos, e as práticas quotidianas numa amostra da população da freguesia da Alcântara. Instituto Superior de Ciências Sociais e Políticas: Lisboa (2009).

[47] Viegas V Azeiteiro UM Dias JA Alves F. Alterações climáticas, perceções e racionalidades / climate change, perceptions and rationalities. Rev Gest Cost Integ. (2014) 14:347–63. doi: 10.5894/rgci456

[48] MAXQDA (2024). All-in-one qualitative & mixed methods data analysis tool. Available at. (https://www.maxqda.com/)

