# Supplementary material for: “We are not ready for this”: physicians’ perceptions on climate change information and adaptation strategies - qualitative study in Portugal
Source: Front Public Health. 2024 Dec 17;12:1506120. doi: 10.3389/fpubh.2024.1506120 (PMC11685147; doi:10.3389/fpubh.2024.1506120)
Supplement: Supplementary file 1 [file Data_Sheet_1.pdf]

“*We are not ready for this*”: Physicians' perceptions on climate change information and adaptation strategies - Qualitative Study in Portugal.

Guião Entrevista semiestruturada

Semi-structured interview Script (In Portuguese)

Objetivo 1: Perceber qual consideram ser o seu papel ao nível da educação para a saúde (em particular os impactos das AC e degradação ambiental na saúde).

1. Acha importante que os médicos abordem as questões AC e os seus impactos na saúde com os seus pacientes?
   1.1 Porquê (quer seja sim ou não)
   1.2 Como é que acha que o podem fazer?

2 Já alguma vez abordou numa conversa com o seu paciente o assunto das AC?
   2.1 Se sim porque o fez? Que conselhos deu para alterar hábitos que possam melhorar a saúde e mitigar os efeitos das AC?
   2.2 Se não, porquê? Está a planear e fazê-lo no futuro?

3 Pensa que os médicos podem influenciar os seus pacientes a alterar alguns dos seus comportamentos, com o cobenefício para a sua saúde e para o ambiente?
   3.1 Sim e Não, porquê?
   3.2 Caso afirmativo, como?

4 Pensa que os médicos podem influenciar o público e os decisores políticos a serem mais pró-ativos a tomar medidas mais eficazes no combate às AC?
   4.1 Como? Não Porquê?

5 E no local de trabalho, pensa, que os médicos podem ter um papel ativo, encorajando os responsáveis no seu local de trabalho a serem tão ambientalmente sustentáveis quanto possível?
   5.1 Como?

Objetivo 2: Perceber a informação que os médicos têm sobre as políticas das AC referentes à saúde em Portugal e se elas são suficientes. E compreender se estes profissionais acreditam que as suas ações poderão influenciar as políticas locais e nacionais de adaptação às alterações climáticas no setor da saúde.

6. O ministério da saúde ou a delegação regional de saúde ou outros organismos dão informação sobre as doenças ou seu agravamento, relacionadas com as AC?
   6.1 Esta informação é importante?

7. Existem procedimentos instituídos? A que nível? Quais são? E na sua opinião como deveria funcionar?
   7.1 Acha que devia haver esse tipo de informação?

8. Na sua opinião, os serviços de saúde estão preparados para lidarem com os agravamentos que as AC trazem para a saúde humana? Porquê?
   8.1 Qual a sua sugestão para que se possa melhorar essa questão

9. Na sua opinião, quem pensa que é responsável por encontrar soluções para as consequências das AC?
