# Supplementary material for: “We are not ready for this”: physicians’ perceptions on climate change information and adaptation strategies - qualitative study in Portugal
Source: Front Public Health. 2024 Dec 17;12:1506120. doi: 10.3389/fpubh.2024.1506120 (PMC11685147; doi:10.3389/fpubh.2024.1506120)
Supplement: Supplementary file 2 [file Data_Sheet_2.pdf]

Sociodemographic Characterization Survey

1. Sex

F ☐ M ☐ **2. Age** ________

3. Civil status

Single ☐ Married ☐ Divorced ☐ Widowed ☐

Other ______________________________

4. Academic Qualification

Degree ☐ Master's Degree ☐ PhD ☐ Post-Doc. ☐

Other ______________________________

5. Medical Specialty ______________________________

6. What year did you graduate from medical school? ____________________

7. What university did you graduate from?

____

8. How many years have you been practicing medicine? ____________________

9. Where do you work (Parish/County)?

____

10. Your workplace is:

Hospital ☐ Clinic ☐ Healthcare Center ☐ Consultancy ☐

Other ______________________________

11. The typology of your workplace is:

Private ☐ Public ☐ Mixed ☐

Other ______________________________
