# Supplementary material for: “We are not ready for this”: physicians’ perceptions on climate change information and adaptation strategies - qualitative study in Portugal
Source: Front Public Health. 2024 Dec 17;12:1506120. doi: 10.3389/fpubh.2024.1506120 (PMC11685147; doi:10.3389/fpubh.2024.1506120)
Supplement: Supplementary file 3 [file Data_Sheet_3.pdf]

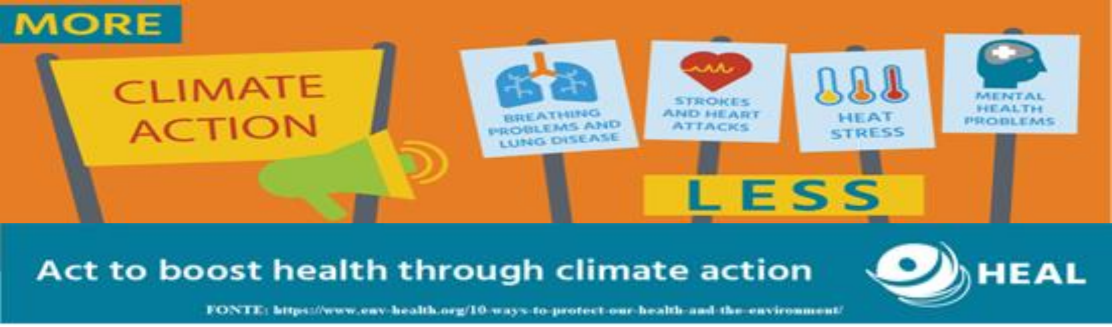

Research Project

We are asking for your participation in this research project; the main aim is to understand whether physicians identify themselves as critical players in raising awareness among their patients about the impacts of CC on health.

Your collaboration in this project consists of a single interview in which you will answer questions about the role of physicians in education on the impacts of climate change health and policies of climate change health impacts.

- The location of the interview is up to you: It can be online, via ZOOM, or face-to-face at a location of your choice.

- The interview will be recorded and transcribed and will only be used in the context of this study and related scientific publications.

- •All data collected during the interview will be treated confidentially, and your name will not appear at any point during the interview or in the data processing results.

- •Any reports or publications resulting from this study will be based on the collective and anonymous processing of the data of all the physicians participating in this project.

- If you have any questions about your rights as a participant, you can contact the researcher:

Nidia Ponte by Phone: [redacted] or email: [redacted]

SOCIEDADES E SUSTENTABILIDADE AMBIENTAL
CENTRE FOR FUNCTIONAL ECOLOGY
SCIENCE FOR PEOPLE & THE PLANET
Laboratório Associado TERRA
1 2 9 0 UNIVERSIDADE D COIMBRA
Universidade de Coimbra
UNIVERSIDADE ABERTA
www.uab.pt
