# Supplementary material for: “We are not ready for this”: physicians’ perceptions on climate change information and adaptation strategies - qualitative study in Portugal
Source: Front Public Health. 2024 Dec 17;12:1506120. doi: 10.3389/fpubh.2024.1506120 (PMC11685147; doi:10.3389/fpubh.2024.1506120)
Supplement: Supplementary file 4 [file Data_Sheet_4.pdf]

CONSENT STATEMENT

Research Project

I, the undersigned, (full name of participant) ____ I have freely agreed to participate in the study "The Role of Physicians in Health Education on Climate Change" by researchers Nidia Ponte, Fátima Alves, and Diogo Guedes Vidal.

I declare that I have understood the explanation given to me about my participation in the research to be carried out and the study in which I will be included. I was given the opportunity to ask the questions I thought necessary, and I received satisfactory answers to all of them.

I have been informed that my participation will consist of an interview with a sociodemographic characterization and questions related to the health impacts of climate change. In this I would share my thoughts and experiences about the impacts of climate change on health in the interview, mainly about physicians' roles as educators on health and climate change.

I can decide to stop participating at any time during the interview, and any information will be deleted. Also, I was informed that my participation in the interview did not represent any harm or risk to my health, and I didn't get any benefit from it.

I have been assured that all data relating to my identification in this study will be kept confidential and anonymous. Importantly, I retain the right to withdraw from the study at any point, without any personal consequences. Additionally, I can request to review the study's findings at my discretion.

The interview will be recorded and intended solely for later transcription for use in the reports and papers written about this research. Therefore, I authorize the recording of the interview, and the dissemination of the results obtained in the scientific environment, with anonymity guaranteed.

Date: Participant's signature:
